# Multifunctionalized Microscale Ultrasound Contrast Agents for Precise Theranostics of Malignant Tumors

**DOI:** 10.1155/2019/3145647

**Published:** 2019-07-07

**Authors:** Jia-Wei Fu, Yi-Sheng Lin, Sheng-Long Gan, Yong-Rui Li, Yao Wang, Shi-Ting Feng, Hao Li, Guo-Fu Zhou

**Affiliations:** ^1^Guangdong Provincial Key Laboratory of Optical Information Materials and Technology & Institute of Electronic Paper Displays, South China Academy of Advanced Optoelectronics, South China Normal University, Guangzhou 510006, China; ^2^National Center for International Research on Green Optoelectronics, South China Normal University, Guangzhou 510006, China; ^3^Department of Radiology, The First Affiliated Hospital, Guangzhou University of Traditional Chinese Medicine, Guangzhou, Guangdong 510405, China; ^4^Department of Radiology, The First Affiliated Hospital, Sun Yat-Sen University, Guangzhou 510080, China

## Abstract

In ultrasonography, ultrasound contrast agents (UCAs) that possess high acoustic impedance mismatch with the bulk medium are frequently employed to highlight the borders between tissues by enhanced ultrasound scattering in a clinic. Typically, the most common UCA, microbubble, is generally close in size to a red blood cell (<∼10 μm). These microscale UCAs cannot be directly entrapped into the target cells but generate several orders of magnitude stronger echo signals than the nanoscale ones. And their large containment and high ultrasound responsiveness also greatly facilitate to perform combined treatments, e.g., drug delivery and other imaging techniques. So multifunctionalized microscale UCAs appear on this scene and keep growing toward a promising direction for precise theranostics. In this review, we systematically summarize the new advances in the principles and preparations of multifunctionalized microscale UCAs and their medical applications for malignant tumors.

## 1. Introduction

The diagnostic yield of ultrasound imaging, namely, ultrasonography (US), basically depends on the backscattering echo intensity, which is proportional to the change in acoustic impedance between different interfaces with different densities [1]. However, in the inner aqueous environment of human body, the density difference between the lesions and background organs or tissues is not high enough to produce clear contrast image in the ultrasound field. In most cases, some high impedance substances are administered to patients to enhance the echo signals within the highly vascular region by intravenous or local injection for a strong contrast effect. The direct consequences include to highlight tissue borders, such as cardiac chamber and ventricular wall; to provide an alteration in the expected time-dependent or geographic distribution patterns in a tissue of interest, such as a tumor in the liver; and to visualize blood and blood flow within an organ for distinguishing normal from injured or abnormal tissues [2]. So these substances are named as “ultrasound contrast agent” (UCA).

In general, the contrast-enhanced effect of UCA crucially relies on its backscatter intensity (*I*), defined as follows:

(1) $$\begin{array}{c} \frac{I}{I_{0}}∼\frac{1}{9}nV\left[k^{4}r^{6}\frac{{\left(\gamma_{\text{c}}+\gamma_{\text{d}}\cos\;\theta \right)}^{2}}{d^{2}}\right], \end{array}$$

where *I*_0_ is the incident intensity, *n* is the number density of scattering particles, *V* is the scattering volume, *k* is the number of the incident wave (the ultrasound emission frequency), *r* is the radius of the particle, *γ*_c_ is the compressibility term (*γ*_c_ = (*κ*_s_ − *κ*_m_)/*κ*_m_, where *κ*_s_ and *κ*_m_ are the compressibility of the scatterer and ambient medium, respectively), *γ*_d_ is the density term (*γ*_d_ = (3*ρ*_s_ − 3*ρ*_m_)/(2*ρ*_s_ + *ρ*_m_), where *ρ*_s_ and *ρ*_m_ are the densities of the scatterer and ambient medium, respectively), *θ* is the scattering angle (180° for backscattering), and *d* is the distance from the scatterers. In a determined ultrasound field, *I* depends mainly on *r*, *γ*_c_, and *γ*_d_ terms. Hereinto, *I* is in direct proportion with the sixth power of *r*. This means that the larger the bubble, the better the scattering intensity. But too large UCA may block blood vessels, especially blood capillaries.

Hence, most of commercial UCAs possess same size distribution, approximately equal or slightly smaller than the size of a red blood cell (<∼10 *μ*m) but much larger than the molecules and particles used for computed tomographic (CT) and magnetic resonance imaging (MRI) [2, 3]. In this situation, how to improve the density and compressibility differences between UCA and water becomes a key to further increase the backscatter intensity and the resulting imaging quality.

### 1.1. History

In the late 1960s, Dr Charles Joiner, a cardiologist, was performing M-mode echocardiogram—injecting a patient with indocyanine green through a left ventricular catheter, to measure cardiac output using Fick's dye-dilution principle. At this time, he found transient increases in the ultrasound signal from the ventricle after each indocyanine injection. Subsequent research indicated that these increases originated from small bubbles forming at the catheter tip [1, 4, 5]. This finding opens a door of echo-enhancing agents to high quality diagnosis and thus greatly broadens the clinical application of the ultrasound imaging technique.

In 1988, Feinstein found that albumin, a blood component, could improve microbubble stability, and sonication can be applied to produce more stable and size-controllable microbubbles. His methodology eventually resulted in the first pharmaceutical echo enhancer, Albunex™, by Molecular Biosystems (San Diego, CA, USA) [1, 5]. Afterwards, advanced ultrasound scanner technology and contrast agent detection methods (e.g., Siemens' Cadence Pulse Sequencing® mode) keep being developed and make UCA more effective. Nowadays, some of the microbubbles are commercially available, such as Levovist® (Schering AG), Optison® (Amersham), Definity® (Bristol-Myers Squibb), and SonoVue® (Braco), and have been clinically utilized for enhanced ultrasonography in the US, Canada, Europe, Asia, and so on [4, 5].

### 1.2. Architectures

As previously mentioned, size, density, and compressibility, three crucial elements to determine the contrast performance of UCA, also determine architecture design and material choice of UCA.

#### 1.2.1. Bubbles

**Figure 1:**
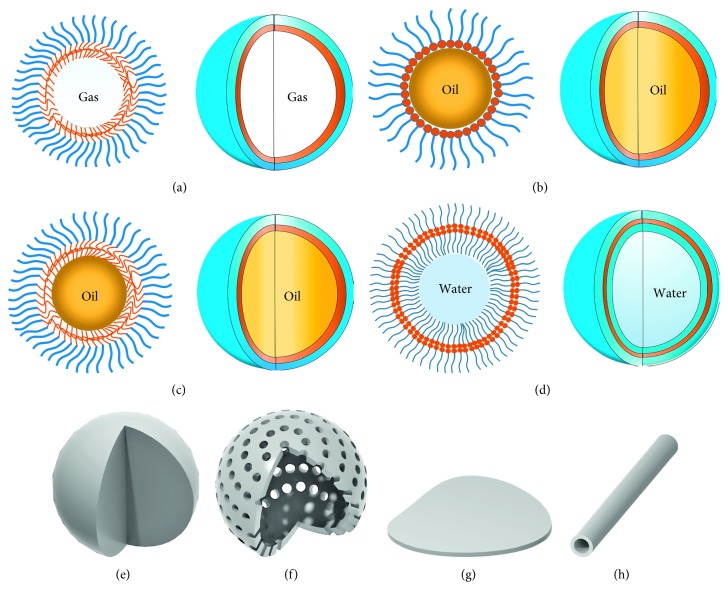
Cross-sectional diagrams and stereograms of ultrasound contrast agents: (a) bubble; (b) emulsion; (c) capsule; (d) vesicle; (e) solid sphere; (f) porous sphere; (g) sheet; (h) tube.

Bubble, as the name implies, is a gas sphere with a thin shell, just as shown in Figure 1(a). Obviously, inner gas core features very higher density difference with ambient aqueous medium to strengthen ultrasonic echoes. And the outer shell comprising “surfactants” make these small bubbles stabilized to prolong the circulation time in body [1–8]. In common, the diameter of most bubbles as UCAs ranges from 3 to 5 *μ*m, which does not allow them to pass through the vascular endothelium. In other words, this provides a pure intravascular ultrasonography [3]. However, under the action of the ultrasound wave, a strong cavitation effect will cause rupture of many bubbles to weaken the contrast effect [9]. Therefore, how to improve the mechanical strength of the bubble remains to be solved.

Recently, some nanobubbles as UCAs have been developed. As already known, nanoscale size contributes the entrapment of these nanobubbles into some organs or tissues with abundant capillaries (e.g., tumors) but also restricts them to achieve a good contrast ratio within the administered regions. And as the size decreases, surface activation energy of these small bubbles becomes more and more high, with the direct result of decreasing stability. It will be very difficult to keep nanobubbles constant. Till now, there are still many doubts about the effectiveness of nanobubbles for ultrasound imaging and others. So, this review will focus on microscale materials for UCAs.

#### 1.2.2. Emulsions and Capsules

Compared to gas, liquid is flowing but must be kept at relatively constant volume. At least, tiny droplets uniformly dispersed into the aqueous phase can behave more stably than bubbles under the ultrasonic action. If the dispersed liquid is a characteristic of high density and strong hydrophobicity (e.g., liquid perfluorocarbon), their aqueous dispersion, namely, emulsion, may also generate obvious echo signals by interacting with the ultrasound wave [10, 11].

Emulsion is a metastable oil-in-water system (Figure 1(b)). According to the size scale of emulsion droplets, ultrasound contrast emulsion can be divided into microemulsion and nanoemulsion. Despite being stabilized by the outer “surfactant,” emulsion droplets in the aqueous phase aggregate very easily and fuse into bigger droplets. This metastable character of emulsions is bound to hinder the intravascular delivery of contrast droplets. Likewise, stable nanoemulsion as UCAs is rarely reported.

If the core is liquid, the capsule-like architecture is almost the same as emulsion droplet's. The difference of the capsule with emulsion droplet lies on “oil” concentration and structure (Figure 1(c)). Emulsion is usually milky, but capsule solution is semitransparent and also transparent. It indicates that the “oil” concentration of capsule solution is much lower than the one of emulsion in the oil-in-water system. And emulsion does not necessarily have the outer “surfactant” shell, but capsule must be. Just for this, the capsule less crashed and fused, showing high stability. In addition, some solid matters with the physical property totally different from the shell and ambient aqueous phase are also available to the inner filler of capsule.

#### 1.2.3. Lipidosomes and Polymeric Vesicles

Vesicle is a hollow sphere that encloses a volume of the aqueous phase (Figure 1(d)). Hereinto, the internal and outside surfaces of vesicles nearby the ambient aqueous medium are hydrophilic, but the interior of the sphere shell is hydrophilic. The forming of the vesicular architecture originates from the interaction of amphiphilic molecules and internal driving of their self-assembly behavior. In fact, lipidosome is a kind of vesicle that consists of phospholipids, and polymeric vesicles do of amphiphilic polymeric molecules [12, 13]. Some unique features, such as much larger surface area, better biocompatibility, and simultaneous encapsulation of hydrophilic and hydrophobic agents, make them applicable for drug delivery and bioimaging. To ultrasonography, although the loading ability of vesicles is limited, their excellent compressibility and stability may facilitate improving the echo ability themselves.

#### 1.2.4. Solid and Porous Spheres

Most of solid spherical particles without the interior filler (Figure 1(e)) fully depend on their clean and hard interface with ambient water to reflect the ultrasound wave. But their limited compressibility makes them incapable of enhancing these echo signals like other elastic scatterers. By contrast, nanomicelles formed by the self-assembly of amphiphilic molecules possess better compressibility and performance of the loading hydrophobic agent (if the loaded agent is gas, this architecture should be named as self-assembled nanobubbles; if liquid, the nomenclature of the “nanocapsule” is exact). Their deficiencies lay on the nanoscale, being incapable of returning an effective echo.

So another kind of solid sphere, namely, porous sphere (Figure 1(f)), has been developing. The porous structure can obviously enlarge the specific surface area (i.e., acoustic reflection area) of these particles and improve their compressibility. On the contrary, surface and interior pores can absorb many tiny bubbles or droplets, which dramatically contribute to the echo enhancement.

#### 1.2.5. Other Architectures

According to equation (1), as long as the particles dispersed in the aqueous phase own high enough density difference with water and large enough reflection surface, they may work to increase local ultrasound contrast and be considered as UCAs, for example, sheet [14], plate [15], and tube [16]; these irregularly shaped particles (Figures 1(g) and 1(h)) have much larger specific surface area than regular spherical particles with the same volume. Regardless of poor compressibility, their potential loading or encapsulating capability to hydrophobic agents might make up for this shortcoming, even achieve a further optimized contrast effect.

### 1.3. Materials

In equation (1), both the density term (*γ*_d_) and the compressibility term (*γ*_c_) except the *r* term (i.e., the radius of scatterer) are heavily dependent upon the material for constructing UCA. First and foremost, the material must be nonwater soluble. Whatever the nature, the hydrophilic materials after modification or cross-linking can be also available to the UCA matrix with high contrast and stability.

#### 1.3.1. Framework Materials

In general, surfactant is the best choice of framework materials of UCA. As we know, this kind of amphiphilic molecules can greatly increase the surface tension to make bubbles, droplets, and tiny particles stabilized in water. Phospholipids, polymeric phospholipids, amphiphilic biodegradable polymers, and some biocompatible surfactants are involved [4]. Likewise, hydrophobic heads or blocks aggregate on the interface within dispersion, and conjugated hydrophilic tails or blocks extend to the ambient medium, which fully attributes to the spontaneous interaction of hydrophobic and hydrophilic fragments with each interface.

Of course, hydrophilic materials, including protein and hydrophilic polymers, can also form stable echo conjugation by internal cross-linking, to cover unstable micro/nanosized heteroplasmons [4]. The most common protein for UCA is albumin, e.g., GE Healthcare-made Albunex that is the first albumin UCA approved by the US Food and Drug Administration (FDA). Albunex is an air-filling microbubble coated with human serum albumin. During its preparation process, heating is very important to denature the albumin prior to sonication. All of these denatured albumin in multiple orientations hold together to construct the microbubble shell through disulfide bonds between cystine residues.

#### 1.3.2. Interior Fillers

Except solid microspheres and nanoparticles, almost all of UCAs have interior fillers within the enclosed space comprising framework materials. Gas, liquid, and even solid matters totally different from the framework and medium in nature can be encapsulated in all kinds of bubbles, capsules, emulsions, and particles. Although the interior filler of both liposomes and vesicles is water, the hydrophobic substance inside the vesicle shell can also load the abovementioned fillers. Topically, air [17–22], inert gases [23, 24], sulfur hexafluoride (SF_6_) [25], oil [26], and fluorocarbon (including gas and liquid) [27–32] are frequently applied. Hereinto, fluorocarbon is considered as the most suitable filler, whose high water-repellency, thermal stability, chemical inertness, and gas or liquid density can highly help its carrier to achieve strong and long-time intravascular ultrasound contrast [33]. Due to similar reasons, SF_6_ was chosen as a familiar filler of commercial UCA.

### 1.4. Preparation Methods

The first step of UCA preparation is undoubtedly to disperse water-insoluble gas, liquid, or solid matters into tiny “particles.” Compared to conventional stirring, high speed homogenization is much superior to mash those insolubles up to microscale and submicroscale but still lack in high degree of control over the size and uniformity of resulting particles [6]. As a more advanced preparation method, ultrasonic emulsification can provide high-intensity dispersing action via cavitation to obtain nanosized UCA [29]. The pity is that the resulting narrow particle-size distribution is also impossible to achieve significant harmony-enhancement on ultrasound echoes for higher contrast.

Recently, some novel preparation techniques with excellent controllability, such as template [34–36] and microfluidic method [37–39], have been developed to gain single-sized UCA. Unlike ordinary microfluidics, Chakraborty and Chakraborty applied those rotated round and round tracks of the lab-on-a-compact-disk as the channel of the microfluidic chip to prepare microbubbles [40], and Stride and Edirisinghe combined the microfluidics technique with coaxial electrohydrodynamic atomization to control the particle size and its distribution of resulting microbubbles [41]. However, the template method is too complicated to be integrated into practical application, and the microfluidic method is only applied to few UCA productions because of the limitation of the chip substrate.

### 1.5. Interaction with the Ultrasound Wave

Almost all UCAs, except solid contrast particles, behave with high complexity under the action of the ultrasound beam. Framework materials and interior fillers enable much better compressibility than soft tissues and organs. Just for this, these insonified UCAs can resonate like a musical instrument and even maximize echogenicity, namely, relative strength of the backscattered signal, near the microbubble resonance frequency once exposed to the ultrasound pulse. For example, clinically applied UCAs with the size of few micrometers have typical resonance frequency within the frequency range of diagnostic ultrasound (1∼10 MHz). This will be very helpful to detect, locate, and distinguish UCA from similar tissue reflectors such as red blood cells [1, 4]. In addition, countless echogenic ultrasound scatters with narrow size distribution, even single size just as mentioned above, will produce amplified harmonics to further enhance echo signals because their close, even same resonance frequency can make them resonate each other [9].

However, during this resonating process, UCA will undergo violent and alternative transform between compression and expansion along with ultrasound frequency until thoroughly disrupted and dissolved (except for solid UCA) [9]. Inevitably, too short retention time in the ultrasonic field becomes a fatal defect of UCA that need urgent solution. Therefore, many scientists have been trying to develop novel framework materials with high compressibility and strength, and other scientists have done to quantitatively measure the mechanical characters of UCA (including shell surface intension and elastic coefficient) for exact evaluation on stability [17, 42–50]. Particularly to the latter, it is very regrettable that no one can plot out an accurate measuring technology on free and elastic micro/nanosized scatter in aqueous solution till now.

### 1.6. Medical Applications

**Figure 2:**
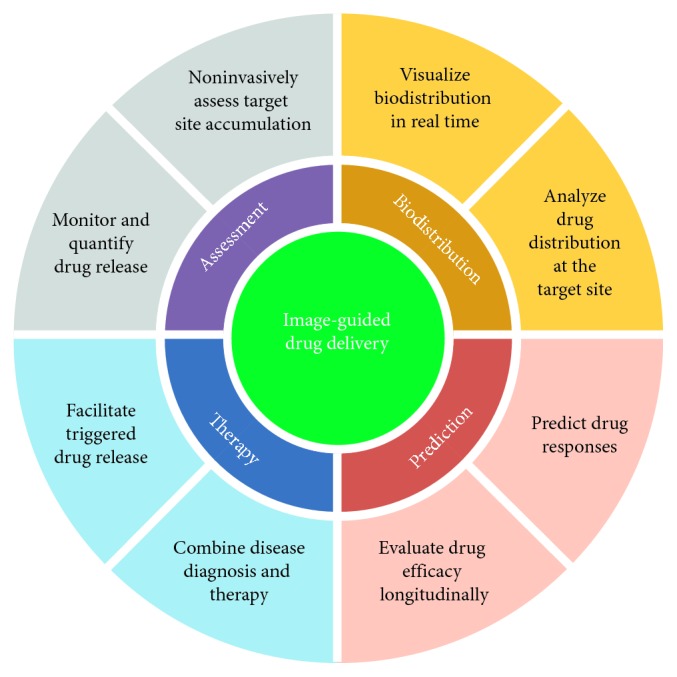
Schematic representation of theranostic applications.

In 2002, the term “theranostic” was first defined as the multimodal combination of diagnostic imaging and therapy [51]. Such a combination of several features into one “package” may overcome undesirable differences in biodistribution and selectivity existing between distinct imaging and therapeutic agents [52]. Once imaging and therapeutic agents are integrated, this coloaded “vehicle” can be visualized by diagnostic imaging to gain real-time information on the diseased tissue, delivery kinetics, and drug efficacy for tuning treatment protocols. Different with traditional “one size fits all,” the “two or more is better than one” with multifunctional theranostic system show more personal treatment according to patient's real-time condition [53–55]. In detail, these characteristic features are exhibited in Figure 2. Given the cavitation effect, UCA can be broken and even disassembled thoroughly under the action of ultrasound. Therefore, it is also viewed as an ideal drug carrier with active control release besides imaging.

According to equation (1), the contrast-enhanced effect of UCA is in direct proportion with the sixth power of *r*. Comparing nanosized UCA to microscale UCA, the average size of microscale UCA is about ten times bigger than nanosized UCA which means the contrast-enhanced effect differs the sixth power of ten. It means the much stronger ultrasound responsiveness does contribute to develop multifunctionalization of microscale UCA. Of course, these “huge” agents are also characteristic of larger capacity and easy preparation. Although nanosized UCA with the capability of cellular uptake attracted more and more attentions [56], microscale UCA is the most popular and effective in clinical applications (Table 1). In this view, we will introduce microscale UCA on three main parts, including malignant tumor diagnosis, therapy, and theranostics. And more sorts will be discussed in detail later.

## 2. UCA for Tumor Diagnosis

### 2.1. Microbubbles

#### 2.1.1. Lipidosome Microbubbles

Lipidosome microbubbles refer to the microbubble shell mainly composed of phospholipids and some lipids, with a bilayer structure which is identical to the cell membrane structure and has a good biocompatibility.

Zhang et al. [57] designed targeted microbubbles (MBs) to assess the local two neuropilin-1 (NRP) targeted peptides (CRPPR and ATWLPPR) concentration and detect small tumors with high sensitivity. The MBs were made of lipids, polyethylene glycol, and peptides (lipo-PEG-peptides), with perfluorobutane (C_4_F_10_) as the core, and the size of MBs is ranging from 1 *μ*m to 5 *μ*m. CRPPR MBs bound to an NRP-expressing cell line 2.6 and 15.6 times more than ATWLPPR MBs and nontargeted (NT) MBs in vitro, respectively, and binding was inhibited both in vitro and in vivo by pretreatment with an anti-NRP antibody. In vivo, tumor echogenicity for the CRPPR-targeted MBs keep 67% of the initial signal after 8 minutes, and it was 8.0 and 4.5 times higher than ATWLPPR and NT MBs without significant effects on tumor blood flow or MB binding. The MBs can show good diagnosis for tumor with CRPPR targeted.

Based on previous work, with the same C_4_F_10_ core and same lipo-PEG-peptides shell, Zhang et al. [58] created a nucleolin- (NCL-) targeted microbubble (F3-conjugated MBs; size: 1∼2 *μ*m). The results showed that F3-conjugated MBs bind to an NCL-expressing cell line at a level 433 times greater than NT MBs because of the greater accumulation of F3-conjugated MBs, with the same dose of contrast agents but using a lower amplitude pulse sequence to reach the same imaging efficacy in the detection of syngeneic murine breast tumors. By targeted NCL, the MBs showed much more cell bind compared to previous work which lead to a better diagnosis efficacy.

#### 2.1.2. Polymeric Microbubbles

As the shell material of UCA, polymers have higher intensity, elasticity, and stability than other common materials, e.g., albumin and phospholipid, and are characteristic of extended circulation time in the body [59]. Particularly, UCA with the controlled size and uniform size distribution can be readily obtained by regulating the material nature of the polymer.

Tsao and Hall reported an enzyme-degradable microbubble using silica and a triethoxysilane end-capped polycaprolactone (SiPCL) with high elasticity for enhanced acoustic responsiveness [60]. The SiPCL microbubble generated over multiple pulses of ultrasound under the mechanical index (MI) of 0.8, while commercial agents already started responding at MIs lower than 0.07 and barely survive more than two ultrasound pulses. Particularly, the microbubble could be efficiently degraded by the lipase enzyme, but it is kept stable without enzyme. This makes them available to controllable and precise ultrasonography.

Song et al. employed the microfluidic flow focusing technology to produce monodispersed microbubbles stabilized by poly(lactic-co-glycolic acid) (PLGA)-PEG as the polymer shell and perfluoropropane (PFP) as the gas core [61]. The size of PLGA-PEG microbubbles can be tightly controlled from ∼600 nm to ∼7 *μ*m with a coefficient of variation less than 4% in size distribution. As a result, the microbubbles displayed the initial gray value smaller than SonoVue's, but their ultrasound signal intensity remained for ∼20 min with only 30% decrease from the initial value, much more stable than SonoVue's.

### 2.2. Other Architectures

Take microcapsules for example. Their ultrasonic properties are significantly dependent on their size and size distribution. Liu et al. prepared several uniform-sized biodegradable polylactone microcapsules by combining a premix membrane emulsification and W/O/W method [62]. Differently, poly(vinyl alcohol) (PVA) was chosen as the core of microcapsules. The results showed that the ultrasound signal intensity and signal duration of PEG-*b*-PLLA microcapsules (∼4 *μ*m) can reach 3.5 minutes continuously. It is considerably stronger and longer than those of commercially available UCAs.

### 2.3. Multimodal Tumor Diagnosis

Ultrasound imaging is one of the most widely used for diagnostic imaging as it is noninvasive. At the same time, it has a relatively low cost and provides for real-time and deep imaging of vital organs [63]. However, ultrasound techniques can produce only low-resolution images of larger structures in a sample, and consequently, higher-resolution analysis of the samples requires the use of an ultrasound contrast-enhancing agent or combining some other higher-resolution imaging modalities that include computed tomography (CT), magnetic resonance imaging (MRI) [64–66], photoacoustic imaging [19, 67, 68], near-infrared (NIR) fluorescence [69], and other imaging modalities.

Benchimol et al. fabricated phospholipid/carbocyanine dye-shelled microbubbles as ultrasound-modulated fluorescent contrast agents to increase imaging depth and spatial resolution of dual-mode imaging [70]. The obtained microbubbles used a gas mixture containing perfluorocarbon as the core and ranged from 1 to 5 *μ*m in diameter. As a result, most microbubbles were able to stably oscillate for more than 10 cycles at pressure of 0.5 MPa for enhancing the imaging effect. In addition, they enabled to extract the optical information at the higher spatial resolution possible with ultrasound under the help of the novel microbubbles. Consequently, the fluorescent microbubbles could be beneficial to the imaging of the deep pathological tissues.

Yang et al. developed multifunctional microbubble probes composed of a nitrogen gas core and a biocompatible PLA shell harboring silver nanoparticles (AgNPs) for integrating ultrasound and optical imaging [63]. Ultrasound imaging studies showed that the presence of AgNPs in the MB improved 70% higher gray scale value. Dark-field microscopy and surface-enhanced Raman scattering microscopy indicated that the optical imaging of AgNP-embedded microbubbles in the living cells was enhanced obviously. In addition, AgNPs were released from the polymer shell following a brief exposure to an ultrasonic field and subsequently taken up by living cells to induce the surface-enhanced Raman scattering for the identifying specific cytoplasmic biomolecules.

**Figure 3:**
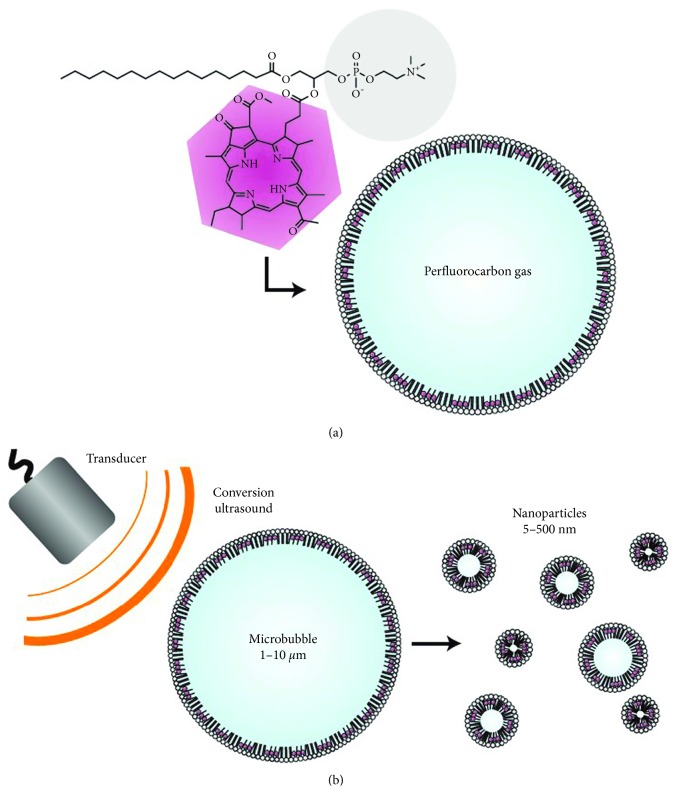
Schematics of porphyrin microbubbles (pMBs) and their micro-to-nanoconversion: (a) pMBs consist of a BChl-lipid shell encapsulating perfluorocarbon gas; (b) conversion of pMBs to porphyrin nanoparticles (pNPs) via sonication with low-frequency, high-duty-cycle ultrasound (conversion ultrasound) (reprinted with permission from the Springer Nature [71]; permission conveyed through Copyright Clearance Center, Inc).

Huynh et al. used porphyrin-lipid to form a shell around a perfluorocarbon gas in the structure of microbubbles (Figure 3) [71]. The encapsulated gas provides ultrasound imaging contrast and the porphyrins in the shell confer photoacoustic and fluorescent properties. On exposure to ultrasound, the microbubbles burst and produced tiny nanoparticles with the result of better optical properties. Particularly, their photoacoustic signals increased from 30 minutes to 2 hours. This conversion is possible in tumor-bearing mice and could be validated using photoacoustic imaging. And it enables the microbubbles potentially used to bypass the enhanced permeability and retention effect during the drug delivery to the tumor.

Barrefelt et al. focused on a ligand-functionalized 99mTc-labeled delivery system that consists of PVA-shelled microbubbles functionalized with different ligands, such as diethylenetriaminepentaacetic acid (DTPA), tihiolated poly(methacrylic acid) (PMAA), chitosan, 1,4,7-triacyclononane-1,4,7-triacetic acid (NOTA), NOTA-superparamagnetic iron oxide nanoparticles (SPION), or DTPA-SPION [64]. The results showed that these microbubbles functionalized by different ligands can be labeled with radiotracers and utilized for computed tomography (CT)/single-photon emission CT (SPECT) imaging, while the incorporation of SPION in microbubble shells enables imaging using MRI. Thus, it may effectively increase the usefulness of multimodality in preclinical and clinical practice by conjugating different ligands.

Xu et al. adopted a premix membrane emulsification method to prepare uniform PEGylated PLGA microcapsules with SPION embedded shell (Fe_3_O_4_@PEG−PLGA MCs) for ultrasound/magnetic resonance (MR) bimodal imaging [65]. In vitro and in vivo trials demonstrated that Fe_3_O_4_@PEG−PLGA MCs (∼3.7 *μ*m) with very narrow size distribution (PDI = 0.03) could function as efficient dual-modality contrast agents which yielded excellent ultrasound contrast with 3 minutes longer duration and strong T_2_-weighted contrast enhancement in the kidney, spleen, and liver. It is promising for accurate diagnosis and real-time monitoring of tumor.

Wang et al. filled the clinically used indocyanine green (ICG) with perfluorocarbon gas into biocompatible PLGA microbubbles for more efficient sentinel lymph node imaging [69]. Unlike a simple ICG solution, ICG-PLGA microbubbles effectively protected against the degradation of ICG inside, which enhanced the NIR fluorescence signal and ultrasonographic contrast of popliteal lymph nodes of rabbits. This exhibited the potent synergistic effect as a contrast media to enhance near-infrared fluorescence imaging and ultrasound imaging signals.

Li et al. presented a phase-transition contrast agents for combined photoacoustic and ultrasound imaging, comprising a highly volatile liquid perfluorocarbon core (size: ∼250 nm) and a polypyrrole- (PPy-) doped polymer shell (thickness: 30 nm) [67]. Here, PPy represents a broadband absorber covering the visible and near-infrared ranges. As exposed to an optical or acoustic pulse with sufficiently high intensity, these nanosized droplets vaporized into microbubbles providing a strong increase in imaging sensitivity and specificity. The threshold for contrast agent activation can be further drastically reduced by up to two orders of magnitude.

## 3. UCA for Tumor Therapy

### 3.1. Microbubbles

Besides tumor diagnosis, microbubbles show potential as well for ultrasound-induced time- and space-controlled drug release [72]. For this purpose, microbubbles can be coated with drug-loaded nanoparticles that are associated with the bubble shell by hydrophobic interactions or electrostatically attached [73].

#### 3.1.1. Lipidosome Microbubbles

Chen et al. designed a temperature and ultrasound dual-sensitive liposomal microbubble (LB) which was liquid at room temperature but gels at 37°C and pH 7.4 [74]. After modified by *N*-cholesteryl hemisuccinate-*O*-sulfate chitosan (NCHOSC), these LBs were further fabricated into the biocompatible chitosan/glycerol phosphate (CS/GP) thermosensitive gel. The obtained LBs with the diameter of ∼950 nm presented a high encapsulation efficiency (∼90%) and excellent stability of curcumin. Under the action of ultrasound, the loaded drug was released faster to 85%, and the antitumor efficacy in vivo was most efficiently improved by ultrasound suppressed tumor growth.

Yan et al. investigated a feasibility of using paclitaxel- (PTX-) liposome-microbubble complexes (PLMC) with the core of perfluoropropane (C_3_F_8_) for ultrasound-triggered targeted chemotherapy against breast cancer [73]. Here, PTX-liposome (PL) was conjugated on the microbubble surface through biotin-avidin linkage. As a result, 74.39% of the entrapped PTX were released under ultrasound exposure. In control mice after implantation of the T1-derived tumor for 22 days, treatment with PLMC and ultrasound resulted in a more significant 70% reduction in tumor volume (from 1233.47 ± 125.53 to 360.01 ± 131.24 mm^3^). Clearly, the drug release efficiency of PLMC is not high enough but leads to a higher tumor therapy efficacy.

Escoffre et al. constructed a liposome microbubble containing a C_4_F_10_ gas core and a lipid shell coupled with doxorubicin (DOX) by covalent linkages [59]. Its imaging and therapeutic properties were evaluated with the U-87 MG cells. The results showed that a sufficient ultrasound signal was observed and imaged in real time as well as being tracked *in vivo*. And the combination of ultrasound and the DOX-loaded microbubbles induced 4-fold decrease of cell viability compared with the controls, which was correlated to the ultrasound-triggered release of DOX.

#### 3.1.2. Polymeric Microbubbles

The stability and the functionality are crucial to the potential therapeutic applications of drug-loaded microbubbles. They can be improved by surface modification of polymers [21].

Villa et al. developed PVA-based and DOX-loaded microbubbles reacting with a galactosylated chitosan complex [75]. This PVA microbubble was able to localize and release the drug to HepG2 hepatocarcinoma cells overexpressing asialoglycoprotein receptors. But its selectivity and bioadhesive properties for the tumor were influenced by the degree of galactosylation rather than the normal fibroblasts. Based on the functionalized ligands, the cellular uptake of DOX loaded in the functionalized microbubbles was higher in HepG2 than the one in normal fibroblasts. It did not over express the asialoglycoprotein receptors and resulted in the cytotoxicity increasing in HepG2 cells.

### 3.2. Other Architectures

**Figure 4:**
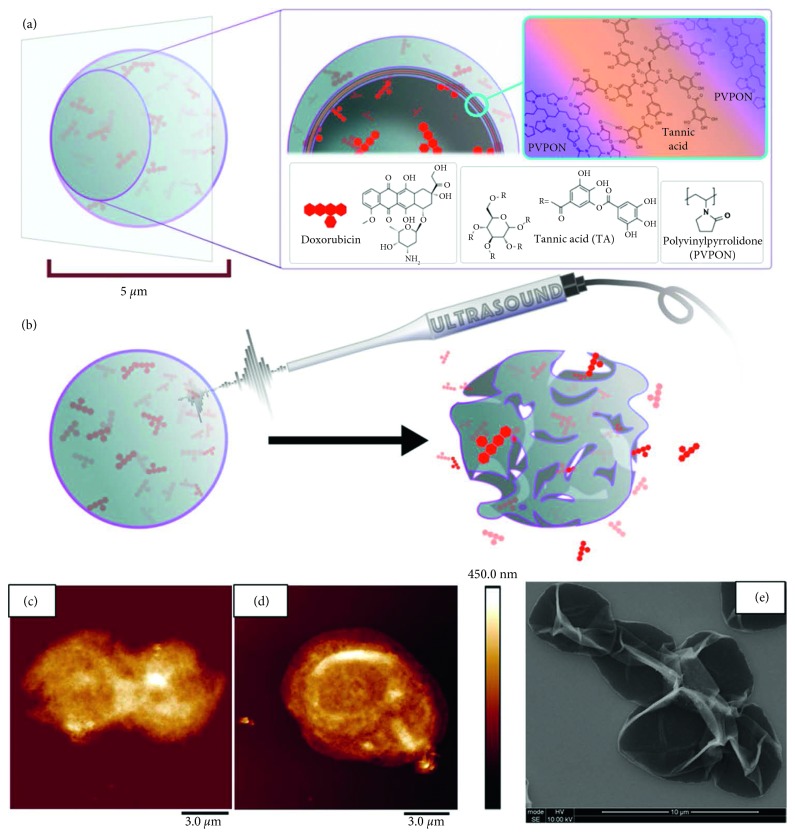
(a) Schematic illustration of hydrogen-bonded TA/PVPON multilayer capsules loaded with doxorubicin (DOX) (inset shows capsule cross section and PVPON and TA molecular structures). (b) Schematic representation of US-triggered capsule perforation/destruction followed by DOX release from the capsule. AFM images of (c) (TA/PVPON-58)_4_ and (d) (TA/PVPON-1300)_4_ capsules. (e) SEM image of (TA/PVPON-58)_15_ capsules [76] (reprinted with permission from the American Chemical Society [76]; permission conveyed through Copyright Clearance Center, Inc).

Chen et al. reported a theranostic polymer microcapsule composed of hydrogen-bonded multilayers of tannic acid (TA) and poly(*N*-vinyl-pyrrolidone) (PVPON) for high imaging contrast and DOX delivery upon low-power diagnostic or high-power therapeutic ultrasound irradiation (Figure 4) [76]. These capsules exhibited excellent imaging contrast in both brightness and harmonic modes and prolonged contrast over six months, much superior to commercially available microbubbles. In vitro studies demonstrated that 50% DOX release from these ultrasound-treated capsules induced 97% cytotoxicity to MCF-7 human cancer cells, while no cytotoxicity was found without the treatment. Furthermore, it was found that, their imaging contrast can be controlled by varying the number of layers, polymer type, and polymer molecular weight.

Do et al. investigated the antitumor potential of combining ultrasound with DOX-loaded PLGA microspheres [77]. An in vitro release assay testified an ability of ultrasound to regulate the release kinetics of DOX from DOX-loaded PLGA microspheres (increase in rate of release: ∼12%). And this treatment could significantly extend survival (mean survival (MS) = 22.1 days) compared to untreated mice (MS = 10.4 days).

### 3.3. Multimodal Therapy

#### 3.3.1. Chemotherapy

Geers et al. constructed a liposome microbubble with a C_4_F_10_ core and a lipid shell conjugating with anti-N-cadherin [72]. The results indicated that such microbubbles can indeed bind to N-cadherin at the surface of HMB2 cells, to deliver the model drug into the N-cadherin-expressing cells due to the ultrasound exposure.

Fan et al. modified 1,3-bis(2-chloroethyl)-1-nitrosourea (BCNU)-loaded microbubbles with vascular endothelial growth factor- (VEGF-) A ligand (VEGF-BCNU-MBs) for antiangiogenic targeting [78]. In the rat model, this novel VEGF-BCNU-MBs significantly further enhanced targeted drug release and reduced tumor progression. And the combination of focused ultrasound exposure and VEGF-BCNU-MBs not only functioned to open the blood-brain barrier (BBB) but also improved tumor-specific targeting, local chemotherapeutic agent delivery, and circulation in the glioma, as well as weaken acute liver accumulation.

Pu et al. synthesized luteinizing hormone releasing hormone (LHRH) receptor-targeted and PTX-loaded lipid microbubbles (TPLMBs) for tumor-specific therapy [79]. Hereinto, PTX encapsulation efficiency reached 73%. After intraperitoneal injection of TPLMBs in the nude mice with human ovarian cancer xenograft, these microbubbles combined with the cancer cells effectively and yielded a superior therapeutic outcome, especially a selective cytotoxicity and apoptosis effect (55.94 ± 8.94%) on A2780/DDP cells. Moreover, immunohistochemical analyses of the dissected tumor tissue further confirmed the increased tumor apoptosis and reduced angiogenesis.

#### 3.3.2. Gene Therapy

Gene therapy may offer a new cancer treatment strategy [80]. Particularly, ultrasound exposure can significantly enhance gene transfection, which greatly contributes to gene therapy.

Typically, the study reported by Li et al. established an effective method to achieve more efficient nonviral gene transfer to stem cells [80]. They employed the synergistic effect of ultrasound combining with microbubbles and polyethylenimine (PEI) to increase DNA transfection efficiency in bone marrow stromal cells (BMSCs). Take a contrast microbubble containing hepatocyte growth factor (HGF) tagged with enhanced green fluorescent protein (pEGFP-HGF) as an example. While exposed the mixture of BMSCs, microbubbles, and PEI : DNA complex to ultrasound, the gene transfection was enhanced efficiency by nearly 38 times compared to the controls. This is the first time that ultrasound plus microbubble in combination with PEI transfection was applied in the stem cell field.

Noble et al. injected plasmid DNA/microbubble complexes with a phospholipid shell into a portal vein (PV) segmental branch and occlusion of the inferior vena cava (IVC), to facilitate DNA uptake and for evaluation on the treatment effect and gene expression [81]. Using an ultrasound amplifier with 15 kW capacity, a 692-fold increase of gene expression was achieved at 2.7 MPa compared to the controls. Similarly, *in vivo* incorporation of plasmid DNA (pDNA) into liver cells was also increased through nonviral gene therapy combined with ultrasound-targeted microbubble destruction for treating hemophilia.

Endo-Takahashi et al. loaded pDNA in the C_3_H_8_ bubble liposomes (p-BLs) that was made of three cationic PEG-modified liposomes for combined ultrasound imaging and gene transfection [82]. Naturally, both of imaging and transfection effect were enhanced to a certain degree with the presence of the p-BLs. Hereinto, the p-BLs containing 1,2-stearoyl-3-trimethylammonium-propane (DSTAP) was the most stable and effective among three types of p-BLs. Furthermore, the p-BLs containing DSTAP was clearly detected using diagnostic ultrasound after systemic injection and powerfully delivered bFGF-expressing pDNA using therapeutic ultrasound.

Sun et al. explored the diverse effects of microbubbles by increased microbubble shell acyl chain length (RN18) and addition of positive charge (RC5K) for greater DNA associability [83]. Compared to clinical Definity®, these microbubble types obviously facilitated transfection of luciferase and GFP reporter plasmid DNA *in vitro* and *in vivo*. Particularly, both RN18 and RC5K were more efficient than Definity®. The cationic RC5K even induced 267-fold transgene expression compared to ultrasound only control.

#### 3.3.3. Phototherapy

Phototherapy has emerged as an optional alternative to conventional chemo- and radiation-based therapies in the treatment of certain cancers. In general, it is divided into two categories: photodynamic therapy (PDT) and photothermal therapy (PTT). PDT involves the simultaneous use of sensitizing drug, light, and molecular oxygen to generate singlet oxygen and other reactive oxygen species (ROS) that result in cytotoxic effects [84]. With the existence of photothermal agents, PTT can effectively absorb external optical stimulus with the tissue-transparent near-infrared (NIR) region (700∼1100 nm) to ablate tumor cells without systemic effects [85].

Typically, Zha et al. constructed PPy hollow microspheres (PPyHMs) with high dispersibility and good stability [85]. These PPyHMs consistently sustained the echo signals for no less than 5 minutes and ablated the tumor completely within 2 weeks under the irradiation of the NIR laser light in a U87-MG tumor mouse model. It showed that this echogenic photothermal agent can act as an efficient theranostic agent not only to enhance ultrasound imaging greatly but also to present excellent photohyperthermic effects.

McEwan et al. investigated rose bengal (RB) sensitizer-decorated, oxygen-loaded, and lipid-stabilized microbubbles (OxyMB-RB) for sonodynamic therapy based on a pancreatic cancer model (BxPc-3) [84]. *In vivo* experiments showed that the mice bearing BxPc-3 tumors were treated with the OxyMB-RB conjugate and ultrasound for five days, following by a 45% reduction in tumor volume. As only treated with the conjugate, the tumor volume in mice increased by 180% over the same time period. Apparently, the combination of oxygen carrying with an ultrasound-responsive therapeutic sensitizer exhibited enhanced sonodynamic activation and a nice *in situ* PDT therapy for tumor.

**Figure 5:**
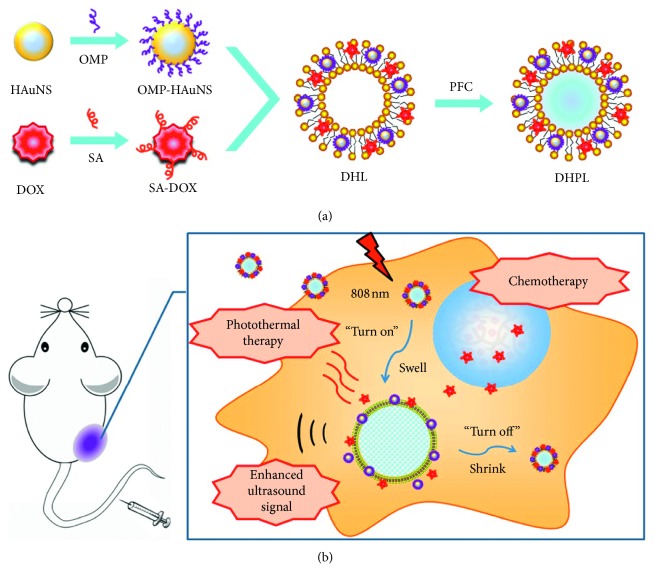
Construction and the antitumor mechanism of DHPL: (a) construction procedure of DHPL; (b) antitumor and ultrasound imaging mechanism of DHPL (reprinted with permission from Copyright Clearance Center, Inc [86]).

Li et al. reported a phase-transition UCA, constructed by coencapsulation of perfluorocarbon with low boiling point, hydrophobic-modified hollow gold nanospheres (HAuNS)(octadecyl 3-mercaptopionate-conjugated HAuNS, OMP-HAuNS), and DOX (stearic acid-conjugated DOX, SA-DOX) into the liposomes (Figure 5) [86]. Under NIR laser irradiation, the generated heat was able to trigger DOX release as well as the gasification of liquid perfluorocarbon. As a result, a significant particle swelling from ∼200 nm to ∼1.2 *μ*m leaded an enhanced ultrasound signal within 5 minutes and almost all DOX release (95.57%) after 8 hours. And the tumor temperature also reached over 41°C within 2 minutes.

## 4. Multifunctionalized UCA for Tumor Theranostics

### 4.1. Microbubbles

#### 4.1.1. Lipidosome Microbubbles

For brain-tumor treatment, Fan et al. proposed DOX and SPION-loaded microbubbles (DOX-SPIO-MBs) to concurrently open the BBB upon focused ultrasound exposure for precise drug delivery [87]. Of course, they acted as dual MRI and UCA and allowed magnetic targeting (MT) to enhance drug delivery. After injection into the rat glioma model, DOX-SPIO-MBs increased local SPION deposition in tumor regions by 22.4% and stably provided significant superparamagnetic/acoustic contrast for imaging and MT.

**Figure 6:**
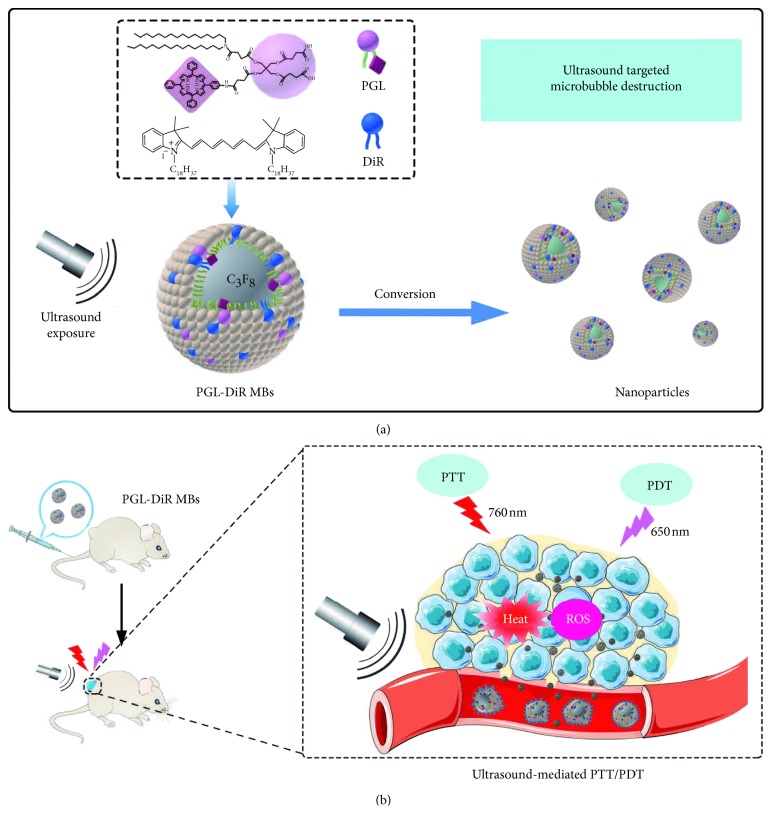
Schematic illustration of PGL-DiR MBs: (a) structure and conversion of the PGL-DiR MBs into nanoparticles when exposed to sufficient ultrasound; (b) ultrasound-mediated tumor-specific combined photothermal and photodynamic therapy (republished with permission from the WILEY-VCH Verlag GmbH & Co. KGaA, Weinheim [88]; permission conveyed through Copyright Clearance Center, Inc).

Xu et al. designed multifunctional microbubbles composed of 1,1-dioc-tadecyl-3,3,3,3-tetramethylindotricarbocyanine iodide (DiR) and porphyrin-grafted lipid (PGL) (PGL-DiR MBs) for ultrasound-mediated combined PTT and PDT and ultrasound/NIR fluorescence bimodal imaging (Figure 6) [88]. Here, DiR functioned as a photothermal agent for photoablation of tumor and a contrast agent to enhance NIR fluorescence imaging. And PGL acted as a photosensitizer for PDT. Particularly, PGL-DiR MBs exhibited remarkably high drug-loading contents (5.8% PGL and 10.38% DiR) and stable drug codelivery. Upon exposure to ultrasound, *in situ* conversion of PGL-DiR MBs into nanoparticles resulted in a 5-fold increase of fluorescence intensity in tumor compared with PGL-DiR NPs, indicating the enhanced tumor accumulation and drug cellular uptake. And PGL-DiR MBs also exhibited complete tumor ablation without recurrence *in vivo*, while PGL-DiR NPs did only 72.6% tumor growth inhibition at the same dose.

**Figure 7:**
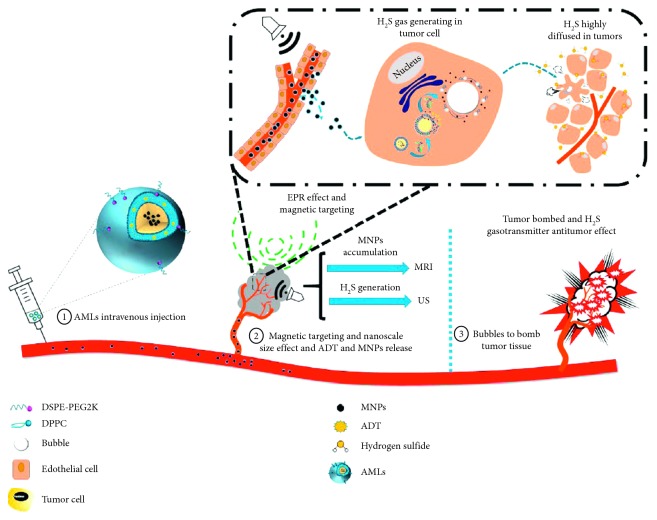
Concepts and schematics of AMLs and their nano- to microconversion for US/MR dual modal imaging and the spatiotemporal bombed combination tumor accurate therapy (republished with permission from the American Chemical Society [89]; permission conveyed through Copyright Clearance Center, Inc).

Differently, Liu et al. [89] reported a stimuli-responsive anethole dithiolethione- (ADT-) loaded magnetic nanoliposome (AML) delivery system (Figure 7). Here, hydrogen sulfide (H_2_S) prodrug was doped in the lipid bilayer, and SPION was encapsulated inside. For *in vivo* applications, after preferentially targeting the tumor tissue spatiotemporally navigated by an external magnetic field, the nanoscale AMLs intratumorally converted into microsized H_2_S bubbles. Moreover, the intratumoral generated H_2_S bubbles can be imaged by real-time ultrasound imaging and further burst to ablate the tumor tissue once exposed to higher acoustic intensity.

#### 4.1.2. Polymeric Microbubbles

Niu et al. prepared SPION and DOX-coencapsulated PLGA microbubbles filled with perfluorocarbon gas (MPMBs) for both tumor lymph node imaging and therapy [90]. *In vivo*, these MPMBs enhanced tumor lymph nodes signals. But after plus sonication treatment on the MPMBs, the tumor proliferation index, microblood vessel density, and microlymphatic vessel density were shown consistently the lowest compared to controls.

#### 4.1.3. Protein Microbubbles

Liu et al. developed a remotely triggered drug vehicle comprising a sulfur hexafluoride (SF_6_) gas core, albumin-shell, camptothecin loaded in the shell, and superparamagnetic micelles self-assembled with carboxymethyl hexanoyl chitosan (CHC) and SPION on the shell surface for multimodal imaging [91]. The results showed that both *in vitro* ultrasound contrast and MRI were enhanced due to the decoration of superparamagnetic micelles. In addition, the release of the loaded anticancer agent could be triggered by therapeutic ultrasound.

**Figure 8:**
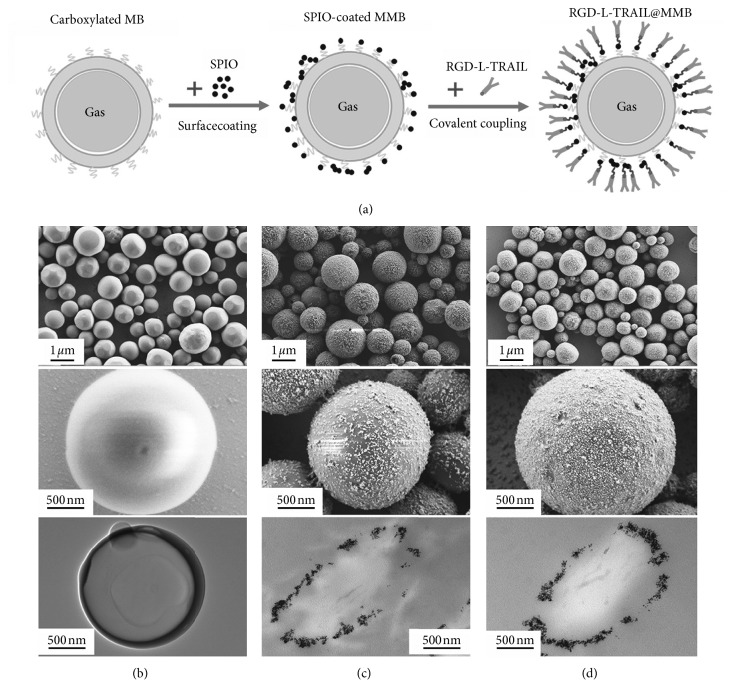
(a) Schematic diagram to show the construction of RGD-l-TRAIL@MMBs. SEM and TEM images of (b) MBs, (c) MMBs, and (d) RGD-l-TRAIL@MMBs (republished with permission from the WILEY-VCH Verlag GmbH & Co. KGaA, Weinheim [92]; permission conveyed through Copyright Clearance Center, Inc).

Duan el al. fabricated cancer-targeting magnetic microbubbles (RGD-l-TRAIL@MMBs; Figure 8) [92]. First, magnetic iron oxide nanoparticles (*γ*-Fe_2_O_3_) were chemically conjugated on the microbubble surface. And then an antitumor targeting fusion protein, arginine-glycine-aspartic acid-l-tumor necrosis factor-related apoptosis-inducing ligand (RGD-l-TRAIL), was precisely bonded with magnetic microbubbles. So RGD-l-TRAIL@MMBs was endowed with the multigradient cascade targeting ability, followed by magnetic targeting, enhanced permeability, retention effect, and regulated targeting. All these effects obviously resulted in high cancerous tissue targeting efficiency. Due to the highly specific accumulation of RGD-l-TRAIL@MMBs in the tumor, the accurate diagnostic information of tumor can be obtained by dual ultrasound and MRI. As an anticancer agent, the TRAIL molecules were also effectively entrapped into the cancer cells by nanoparticles and RGD-mediated endocytosis for the tumor cell apoptosis.

### 4.2. Microcapsules

Jin et al. fabricated Au@PLA-(PAH/GO)_2_ microcapsules by introducing gold nanoparticles into PLA microcapsules using the double-microemulsion method. And then graphene oxide deposited onto the microcapsule surface via electrostatic layer-by-layer self-assembly [93]. It was proved that the microcapsules can serve as a contrast agent to simultaneously enhance ultrasound imaging (more than 5 minutes) and X-ray CT imaging (838 Hounsfield units) both *in vitro* and *in vivo*. The *in vivo* therapeutic examinations indicated that the microcapsule behaved with an effective PTT effect to induce a temperature elevation of 24.4°C. Clearly, the combination of real-time ultrasound with 3D CT through such a microcapsule agent is very helpful for accurately interpreting the obtained images, identifying the size and location of the tumor, as well as guiding and monitoring PTT.

Li et al. fabricated multifunctional theranostic PLA microcapsules with SPION loading and surface functionalization of graphene oxide [94]. The experiments proved that these microcapsules served well as contrast agents to simultaneously enhance ultrasound, MR, and photoacoustic imaging. Upon NIR laser irradiation, the photothermal effect can trigger the temperature elevation of 24°C to efficiently kill cancer cells and even be obviously intensified by applying an external magnetic field.

Zha et al. prepared both PPy nanocapsules (size: 280.4 ± 79.0 nm) and microcapsules (size: 1.31 ± 0.45 mm) with liquid perfluorooctylbromide (PFOB) core [95]. Apparently, the gasification of PFOB leaded to an enhanced ultrasound imaging. And under NIR laser irradiation, a temperature elevation of 26.1°C was obtained to complete photothermal ablation of tumor cells without inducing any significant side effect.

Yan et al. prepared a hematoporphyrin monomethyl ether- (HMME-) loaded PLGA microcapsule (HMME/PLGA) by double emulsion evaporation [68]. It not only functions as efficient contrast agent for ultrasound and photoacoustic imaging but also as synergistic agents for high intensity focused ultrasound (HIFU) ablation. The results showed that the HMME/PLGA microcapsules remarkably inhibited the SKOV3 cell proliferation and even completely killed these cells. Particularly, the greatly enhanced HIFU ablation effects were observed on ovarian cancer in nude mice by the HMME-mediated sonodynamic chemistry therapy (SDT).

### 4.3. Microspheres

Yang et al. developed biodegradable yolk-shell Fe_3_O_4_ @PFH@PMAA-DOX microspheres as ultrasound/MR dual-modality imaging contrast agents and drug delivery system [96]. This nanosystems consisted of poly(*γ*-glutamic acid)- (PGA-) stabilized Fe_3_O_4_ nanoclusters as the magnetic core and disulfide cross-linkage poly(methacrylic acid) (PMAA) as the functional shell. Meanwhile, ultrasound-sensitive perfluorohextane (PFH) was further introduced into the inner cavities of yolk-shell microspheres, and DOX did into the PMAA shells (loading capacity: 15.4 wt%; loading efficiency: 91 wt%). Here, the loaded DOX was controllably released under pH, redox, and ultrasound multistimuli with the result of effective cancer cell apoptosis. The human pancreatic carcinoma cells viability test showed that more than 90% cells were killed at a concentration of DOX in Fe_3_O_4_ @PMAA- DOX at 1.0 *μ*g/mL after treating 48 hours.

## 5. Conclusions

In summary, we comprehensively introduced the background knowledge and new advances in multifunctionalized microscale UCAs and their medical applications for malignant tumors. At the same time, some novel diagnostic or therapeutic technologies, e.g., photoacoustic imaging, gene therapy, and phototherapy, are also continuously integrated into this “theranostic” system. Particularly, many studies displayed outstanding theranostic effects in *in vivo* experiments for malignant tumors. Although nanotechnology started a revolution in biomedical pharmaceutics, all these also pointed up the high efficiency of the microscale contrast agent. Given the basic rule of ultrasonography, this cannot be still replaced with the nanoscale agent, especially to UCA. So we believe that microscale UCAs have huge and promising potentials for multimodal tumor theranostics.

## Figures and Tables

**Table 1:** Difference between nanosized UCAs and submicron/microsized UCAs.

	Nanosized UCAs	Submicron/microsized UCAs
Size	(i) <500 nm	(i) >500 nm
Raw materials	(i) High and special requirements on material structure and property	(i) Cheap and widely available
Stability	(i) Easy to aggregate (ii) Easy to collapse under the action of ultrasound owing to high surface energy	(i) No aggregation (ii) Easy to collapse under the action of ultrasound owing to strong oscillation
Loading efficiency	(i) Low loading efficiency limited by the small core size	(i) High loading efficiency that benefits from the large volume
Capability of cell uptake	(i) Easy to be taken by the cell	(i) Very difficult to the cell uptake
Ultrasonic imaging effect	(i) Very weak, especially to blood pool imaging	(i) Very strong
	(ii) Very difficult for clinical application	(ii) Commercial products for clinical application
Multifunctionalization	(i) Practicable	(i) Practicable
